# The Effect of Educational Technology on EFL Learners’ Self-Efficacy

**DOI:** 10.3389/fpsyg.2022.881301

**Published:** 2022-04-13

**Authors:** Ying Zhang

**Affiliations:** Zhejiang Technical Institute of Economics, Hangzhou, China

**Keywords:** computer-assisted language learning, educational technology, self-efficacy, mobile-assisted language learning, L2 education

## Abstract

This paper aimed at investigating the related studies on educational technology and its effect on English as a Foreign Language (EFL) learner self-efficacy. Earlier studies have proved the positive and significant relationship between learner self-efficacy and educational technology use. Investigations have revealed that improving learners’ dynamic mindsets, online interaction, self-assessment, academic knowledge, and positive affectivity can increase learner self-efficacy. Moreover, the provision of the encouraging context can help develop learners’ self-efficacy in technology-supported education. Furthermore, the study presented the implications and future directions of this line of research for different people, such as EFL teachers, teacher educators, and foreign language scholars. The ideas can improve their awareness of learner self-efficacy in technology-supported educational contexts and its role in L2 education.

## Introduction

Self-efficacy is defined as individuals’ views about their ability to engender the desired results (Pajares, 1996). Traditionally, studies have shown that self-efficacy is an important issue in academic achievement for learners with diverse language proficiencies. Therefore, learners’ attitudes over their capability in language learning are significant in decision-making practices. While many studies have been done on the relationship between self-efficacy and its effect on academic achievement, investigations on the realm of leaner self-efficacy continue to develop as educational technologies appear. Educational technologies are regarded as crucial elements of instruction and learning. Technology-supported educational contexts, such as simulations, adaptive instructors, virtual laboratories, video games, computers, and mobile applications, provide some features to improve language learning. Modern technologies have supported foreign language learners to participate in the educational context of language learning (Rassaei, 2017). Lately, numerous investigations have examined the impacts of integrating various technologies in education (Benson and Chik, 2011). The changes in learning contexts, during the COVID-19 pandemic, require studying the influence of technological education on learner self-efficacy.

## Review of Literature

### The Notion of Self-Efficacy

Bandura (1986) defined self-efficacy as “people’s judgments of their capabilities to organize and execute courses of action required to attain designated types of performances” (p. 391). He stated that self-efficacy pinpoints individuals’ self-reliance on their competence to cope with challenging tasks, and to put into practice the prerequisite strategies to be successful in impending situations. Bandura (2011) also stated that individuals’ self-efficacy specifies the way of regulating and managing learning purposes along with persevering in task accomplishment. It also determines the extent of learner resilience and apprehension in coping with difficulties. In order to boost self-efficacy, Bandura (1995) indicated four principal methods, including mastery experiences, vicarious experiences, social persuasion, and physiological and emotional states.

Studies have shown that self-efficacy can be regarded as a convincing basis for arousing positive psychology constructs, such as enjoyment, hope, pride, well-being, grit, resilience, and engagement. Yin (2021) has emphasized the development of EFL learners’ self-efficacy in their academic accomplishments, as it enhances learners’ enjoyment, hope, and pride in educational contexts. Kassem (2021) argued that EFL learners’ enjoyment is significantly associated with accomplishment, self-sufficiency, and self-efficacy. They argued that EFL learner enjoyment is affected by understanding the target language, teachers’ positive approaches, learners’ self-confidence, and positive foreign language context. Fathi et al. (2020) found a significant relationship between self-efficacy and psychological well-being. They mentioned that job satisfaction and work obligation, along with less anxiety or burnout, are the results of individuals with high levels of self-efficacy and well-being. Based on Bandura’s (1986) theory, they justified that individuals’ opinions affect both their performance in tasks and thinking patterns and feelings as the critical constructs of psychological well-being. Sabouripour et al. (2021) also found out that self-efficacy plays the role of mediator in the correlation between EFL learners’ psychological well-being and resilience. They argued that learners’ confidence in their aptitude significantly influences their competence to preserve their emotional and physical health and to adapt to problems in educational contexts.

Moreover, Malureanu et al. (2021) stated that online learning, during the COVID-19 pandemic, significantly affected the relationship between self-efficacy and grit. Han (2021) also pointed out that grit, as a non-cognitive construct, includes passion, perseverance, and self-efficacy, which are theoretically related to goal achievement. Regarding learners’ engagement, Han et al. (2021) stated that self-efficacy acted as a mediator role in the relationship between emotional engagement, academic engagement, and satisfaction. They argued that EFL learners’ emotional and behavioral engagements were affected by their improved self-efficacy. Tsao (2021) found out that self-efficacy is influential in improving learner engagement. He found out that self-efficacy is the only significant variable affecting learner engagement in writing classrooms. Relating to resilience, Cassidy (2015) revealed that learner self-efficacy is significantly correlated with educational resilience. They argued that self-efficacy is influential when learners cope with difficulty, and they mentioned that learners who believe in their skills are motivated and determined in adverse conditions.

Regarding the studies on the relationship between emotional constructs like motivation, and self-efficacy, Niehaus et al. (2012) found out the relationship between self-efficacy, intrinsic motivation, and academic engagement. They found out that the combination of these variables predicts learners’ academic performance. Omari et al. (2018) also proved the correlation between learners’ motivation and self-efficacy. They stated that learners’ academic performance and learning motivation are affected by their self-efficacy and beliefs. Concerning the association between learner self-efficacy and emotional intelligence, Fathi et al. (2021) found a significant reciprocal correlation between these two constructs. They suggested that well-informed teachers who are able to regulate their academic contexts, employ educational practices, and inspire learners to perform assigned activities are competent in emotional regulation when working on their professional affairs. Abdul Rashid et al. (2020), in their study, have shown a significant correlation between earners’ academic self-efficacy and their emotional intelligence. Their self-efficacy impacts emotional intelligence, and it can accelerate learners’ learning process.

With respect to the studies on the relationship between learners’ negative emotions and self-efficacy, Mede and Krairmak’s (2017) study revealed that there is a negative association between learners’ communication apprehension and self-efficacy. They justified their results by adding that low-level self-efficient learners are not able to attain their aims, and this induces anxiety and depression in interaction. In another study, Tuncer and Doğan (2016) have shown the negative relationship between self-efficacy and communication apprehension. They stated that learners with lower levels of self-efficacy think that language learning is more perplexing than it truly is, and this amplifies their nervousness in educational environments. They also contended that anxious students with lower levels of self-efficacy cannot outperform in educational contexts. Farjami and Amerian (2012) found a positive correlation between self-efficacy and positive affectivities like motivation, and they also found a negative correlation between self-efficacy and anxiety.

Some studies have verified a significant association between students’ academic accomplishment and self-efficacy. de Fátima Goulão (2014), in her study, revealed that learners’ self-efficacy is significantly correlated with academic performance. She argued that learners’ belief in their capability triggers their motivation and resilience, which affects persistence in academic achievement. Using Schwarzer and Jerusalem’s (1995) self-efficacy scale, Asakereh and Yousofi (2018) found out that learner self-efficacy is significantly associated with academic achievement. They argued that some reasons for self-efficacy, such as performance accomplishments, vicarious experience, oral encouragement, and emotive provocation, should be emphasized by instructors to enhance learner academic achievement. Bouih et al. (2021) found out that self-efficacious learners have remarkable achievements in academic environments. Furthermore, some studies have shown that EFL learners’ self-efficacy is significantly correlated with their language skills. These investigations have highlighted the role of stimulating learners’ self-efficacy to have proper outcomes in language learning environments (e.g., Chen and Zhang, 2019; Haerazi and Irawan, 2020; Chung et al., 2021).

### Technology in Education and Its Effectiveness

Dusek (2006) defined technology as “the application of scientific or other knowledge to practical tasks by ordered systems that involve people and organizations, productive skills, living things, and machines” (p. 35). Nowadays, technology, with its continuous developments, has changed learning strategies and teaching methodologies (Hollands and Escueta, 2020). Technologies have developed resources, opportunities, and educational contexts, facilitating the formation of self-directed educational practice (Reinders and White, 2011). Learners can experience language learning by using technological aids to meet their own needs. Technology offers numerous opportunities for EFL learners to simply communicate with native speakers in foreign language contexts (Reinders and Benson, 2017). Thus, it is essential that language learners be familiarized with the capability to involve in technology-based language learning (Reinders and Darasawang, 2012). The integration of technology in the educational context has brought promising opportunities for instructors and learners to increase the efficiency of the pedagogical process (Yenkimaleki and van Heuven, 2019). In this respect, Reiser and Ely (1997) defined educational technology as “a field involved in the facilitation of human learning through the systematic identification, development, organization, and utilization of a full range of learning resources and through the management of these processes” (p. 67).

The effect of technologies on learners’ academic achievement is not clear-cut, with some investigations demonstrating indications of improvements (Fonseca et al., 2014), and others exhibiting signs of negative results (Jacobsen and Forste, 2011). However, the use of educational technology is not limited, and it consists of the technology used to support, expedite, and improve education, presentation, and teaching. Zengin and Aksu (2017) stated that education has been affected by technological developments, such as computers, the internet, emails, mobile applications, and digital games. It is generally approved that digital tools are influential in learning achievement. “Using technology such as computer plays a big role as a multi-sensory collection of text, sound, pictures, video, animation, and hypermedia to provide meaningful contexts to facilitate comprehension” (Butler-Pascoe, 1997, p. 20).

Computers and the internet help learners to communicate efficiently in educational contexts. According to Levy (1997), Computer-assisted language learning (CALL) refers to “the study of applications of the computer in language teaching and learning” (p. 1). Some studies have been done on the role of technology in education in learners’ language skills and their academic achievements. Ratnaningsih et al. (2019) found a significant difference in the CALL use in speaking skills. Marzban (2011) investigated the performance of EFL learners in reading comprehension using CALL and the traditional way. He found out that EFL learners using CALL outperformed in reading comprehension scores. In another study, Bensalem (2020) revealed that the incorporation of technological designs like the internet can boost learners’ reading comprehension. He argued that shyness, communication apprehension, and the individualized differences in learning strategies, as the most important problems in traditional classrooms, can be diminished in online classrooms. A more comprehensive description can be found in Taj et al. (2017), showing that email and the internet can develop learners’ L2 reading comprehension and vocabulary knowledge. They argued that digital tools can boost EFL learners’ working memory to retain and recall word meanings. Regarding listening skills, Barani (2011) found out that the designs of educational technology can enhance learners’ listening skills.

Moreover, mobile-assisted language learning (MALL) has provided new prospects for EFL educators to increase collaboration and performance in learning language skills (Godwin-Jones, 2011). Mobile social applications, including Facebook, Twitter, YouTube, Instagram or Flicker; Course Management Service (CMS), Mobile Podcasting, and Automatic Speech Recognition (ASR), are very significant in language performance and skills (Kim and Kwon, 2012). Keezhatta and Omar (2019) found out that MALL materials enhance EFL learners’ reading comprehension. These materials trigger the interest in reading instruction which has constructive implications on developing the learners’ reading skills. Using WhatsApp as a tool in EFL instruction, Alshammari et al. (2018) found out that online EFL learners outperform in reading comprehension. They argued that technology changes teacher-learner rapport, and their roles in an educational context. Wu (2015), in a study, revealed that mobile devices are critical in providing opportunities for language learners’ achievement in vocabulary and writing skills. Read et al. (2021) found out that listening comprehension as a top-down and bottom-up procedure is improved using MALL. They argued that the use of MALL expands the use of language beyond the classroom contexts, giving the learners some chances to develop and modify their information about language use. However, Sadeghi and Dousti (2013) stated that it is not easy to investigate the effectiveness of CALL and MALL on language learners’ performance. They argued that the efficiency of CALL and MALL programs depends on the proper use of the program. Moreover, the efficiencies of CALL and MALL programs are influenced by intervening moderator variables, including the individual differences, task complexity, and the instructional environment.

Educational technology affects and challenges learners’ positive and negative emotions (Guri-Rosenblit, 2018). For example, learners’ grit can be affected by online educational contexts. For instance, McClendon et al. (2017) stated that some non-cognitive factors, such as grit, persistence, and mindsets, are significantly affected by online learning in technological education. They argued that grit, mindset, and persistence increase learners’ engagement in online classes and improve learner achievement. Lan and Moscardino (2019) argued that grit was regarded as an essential component of educational success in a COVID-19 pandemic, and it was considered a psychological procedure that triggered and guided individuals’ activities in online learning contexts. Kosasi and Sulastri (2021) also showed that grit has a key role in affecting academic achievement in virtualized contexts. They argued that grit and knowledge of technology can improve learners’ academic achievement. Furthermore, educational technology can foster learner engagement (Chiu, 2021). Halverson and Graham (2019) provided a theoretical framework for learner engagement. They offer cognitive and emotional scales for estimating learner engagement in online language classrooms. Golonka et al. (2014) asserted that the primary objective of technology is to engage learners and improve social interactions with peers. Khojah and Thomas (2021) found that task designs *via* digital technologies in educational contexts can raise learner engagement in language skills.

Regarding negative emotions, Doğan (2020) investigated the effect of online and traditional learning on learners’ foreign language anxiety. He found that learners are inclined to be anxious in an online context. He found two levels of anxiety among learners: foreign language anxiety and online learning anxiety. He argued that a difference between a learner’s anticipation, experience, and apprehension from technology among learners can justify the reason for learners’ foreign language anxiety and online learning anxiety. Heckel and Ringeisen (2019) found a negative correlation between anxiety and self-efficacy in online language learning. They argued that self-confident learners can operate appropriately when handling a particular online platform, and they feel lower levels of apprehension in online contexts. This review tries to ponder the relationship between technology use in an educational context and learners’ self-efficacy.

### The Role of Educational Technology in EFL Learners’ Self-Efficacy

There is a number of studies that scrutinized the role of educational technology in learner self-efficacy, and the reasons behind the self-efficacy in online contexts. Bates and Khasawneh (2007) argued that learner mindsets are important in the enhancement of self-efficacy in online learning contexts. They argued that learners who have dynamic mindsets and consider online learning ability a flexible and comprehensible skill are self-efficacious ones. In contrast, those who are inclined to have fixed mindsets over online learning ability have lower levels of self-efficacy levels. Lailiyah and Cahyono (2017) found a significant relationship between self-efficacy and the integration of technology into education. They argued that language knowledge and age can mediate the relationship between self-efficacy and technology use. Wu and Yang (2016) also showed that technology-based language learning activities enhance learners’ engagement in self-directed learning and improve learner self-efficacy. However, Peechapol et al. (2018) listed some factors influencing on the improvement of self-efficacy in online learning contexts. They mentioned that online feedback significantly boosts learner self-efficacy. They also asserted that online interaction and engendered trust between peers, and augment learner-self efficacy in online contexts. Moreover, they stated that learner motivation and attitude can trigger higher levels of self-efficacy. In line with Peechapol et al.’s (2018) study, Aysu (2020) found out that learners have more integrative motivation than instrumental motivation in technology-based contexts. He also maintained that motivated learners in online contexts have some features such as “being goal directed, expending effort, being persistent, being attentive, having desires (wants), exhibiting positive effect, being aroused, having expectancies, demonstrating self-confidence (self-efficacy), and having reasons (motives)” (p. 2).

Su et al. (2018) confirmed the significant role of technology-based education in the improvement of learner self-efficacy and suggested that teachers should emphasize learner’s self-assessment in the online educational contexts in order to boost learner self-efficacy. Ningias and Indriani (2021) focused on learners’ points of view about self-efficacy during online education. They found that learners with high levels of self-efficacy are aware of online material, and they explain about them in online contexts. They argued that self-efficacious learners are confident in using some approaches in giving lectures during an online context. They attributed their results to the environment of the classroom, which encourages learners to communicate with their peers. They also mentioned some reasons such as background knowledge, the experience of vicarious, public encouragement, and affective factors which are significant in learners’ self-efficacy during online education. Pantu (2021), in his study, revealed that learner self-efficacy influences academic flow in online learning situations. They argued that independent learning is the key point in online learning contexts and self-efficacious learners can control and manage their learning properly, and perform their online tasks smoothly. Ngo and Eichelberger (2021) also have shown that learners’ level of self-efficacy depends on their comfort levels in using information and communication technologies. They stated that learners’ self-efficacy hinders them from avoiding doing challenging tasks in online contexts. Lian et al. (2021) found the significant role of language learners’ authentic online interaction in the enhancement of self-efficacy. They argued that online interaction can improve learners’ ability to communicate and self-confidence in doing tasks in educational contexts. They suggested organizing technology-based authentic language learning tasks during the COVID-19 pandemic. Rahmania (2020) found a significant correlation between learner self-efficacy and online language learning performance. She argued that the change in the learning conditions, during the COVID-19 pandemic, which coerces learners to do online tasks influence their self-efficacy levels. She also stated that the learners’ background knowledge in performing online tasks can affect their confidence and self-efficacy since those learners have the motivation from the beginning of the online course.

Some studies employed the effect of different technology contexts on learner self-efficacy. For example, Namaziandost and Çakmak (2020) found out that females, compared to males, have high levels of self-efficacy in flipped classrooms. They ascribed their outcomes to the high levels of female engagement in language learning, group discussions, teamwork, role play, and problem-solving in flipped educational contexts. Kasuma et al. (2021) also approved the positive perspectives of learners toward flipped learning, as their self-efficacy, positive emotions, and motivation augmented. They argued that motivated and efficacious learners carry out online English tasks through numerous social media applications. They argued that Learners’ experience in flipped learning contexts before online learning increases their efficacy. Moreover, they mentioned that student-centered tasks improve self-efficacy and autonomy in flipped learning contexts. In another context, Tavakoli et al. (2019) integrated task-based language teaching with online learning, and their study revealed that learners’ self-efficacy is affected by task-based online resources. They attributed their results to the novelty of online learning in Iranian contexts and the engagement of learners. They argued that interactive communication enhances self-efficacy and learning performance. In another study, Honarzad and Rassaei (2019) revealed that learners’ self-sufficiency, self-efficacy, and motivation are significantly affected by technology-based language learning tasks. In another different study on educational technology, Balaman (2020) revealed that digital storytelling, as a mixture of narration with multimedia devices, significantly affects learners’ self-efficacy and approaches toward instructive technology. She argued that mastery experience, as a trigger element of self-efficacy, can elucidate the reason for learners’ high scores of self-efficacy. During her investigation, learners’ narration of five distinct stories created a feeling of achievement in this technology-based educational context. This indication of mastery experience ultimately supported self-efficacy in the classroom. Mobile applications create different learning contexts for learners in educational technology designs. Rachels and Rockinson-Szapkiw (2017) studied the role of MALL on elementary learners’ academic accomplishment and self-efficacy. Their study revealed that Duolingo significantly improves learners’ self-efficacy and academic achievement. They supported the use of gamification to enhance learners’ positive emotions for language learning. They argued that gamification produces scaffolding that increases learner self-efficacy.

## Implications and Suggestions for Further Research

This review concluded that learners’ use of educational technology significantly improves their self-efficacy. However, this review showed that learners’ online interaction, academic knowledge, and strategies for measuring their academic quality can increase learner self-efficacy. The aim of this review was to inspect the related literature on the influence of educational technology on learner self-efficacy. It improves the educational knowledge of investigators who are interested in learner self-efficacy and its position in technology-supported educational contexts. Regarding the related literature about the positive role of technology-supported education in learner self-efficacy, it is worth noting that learners should be helped to manage their emotions in educational environments. Learners can improve their self-efficacy by choosing easy-to-difficult authentic digital tools based on their language proficiency level. Teachers can provide a context for learners to perceive peers’ accomplishments in a task. This can boost learners’ self-confidence as they can carry out their activities independently. Teachers can improve learner self-efficacy with reliable interaction and constructive comments to help learners persist in their efforts. Also, being aware of learners’ personality traits may encourage teachers to do their best to increase learner self-efficacy in educational contexts. Thus, L2 instructors are required to talk to learners about their internal and external motivation and ask their problems to improve their positive viewpoints over technology themselves. Furthermore, paying attention to needs analysis should be one of the first principles of teaching in technology-supported education to achieve educational outcomes. Therefore, they need to modify the digitalized materials based on learner competence to reduce technology-based cognitive load, increase learner attention, and build on learner self-efficacy and positive attitudes toward technology.

Instructors can use moderately difficult activities, which can empower learners with low levels of self-efficacy. The activities should not be too difficult to curb learner self-confidence in doing tasks. Teacher support, including scaffolding, assigning sufficient time, decomposing difficult tasks into simple phases, explicating the task in technology-supported education is influential for the enhancement of learner self-efficacy. This can produce an insight of reasonable challenge and equalizes the complexity of technology-supported tasks. Praising and giving feedback to learners are also important for the improvement of learner self-efficacy. Moreover, teachers should not compare the performances of learners with each other. Teachers can provide learners with some strategies such as self-verbalization. For example, they can motivate learners to express the procedure of learning grammatical points or vocabulary aloud and give feedback on their effort. Moreover, teachers can set a cooperative context, rather than a competitive one, to increase learner interaction and scaffolding, which can improve learner self-efficacy. They can also ask learners to write comments about their feelings and progressions in technology-supported contexts. They may also change their methodology by taking self-efficacy into account in their instruction. They can offer warming-up activities for educational contexts and brainstorm learners to increase self-efficacy in online contexts. The projects, lectures, conferences, and workshops may put extra stress on learners and decrease their self-efficacy in technology-supported situations. In order to increase learner self-efficacy, familiarizing learners with questions of the tests can be helpful. Providing a competitive educational context through quizzes augments learner self-efficacy. Unplanned quizzes are predominantly important for stimulating learners with less self-efficacy levels. Teachers can provide learners with some video files like TED videos in online classrooms, and they can discuss the effect of self-efficacy on the improvement of individuals’ performance.

This study has some implications for teacher educators, policymakers, and advisors. In order to improve teacher self-efficacy, teacher educators and mentors can provide a situation in which teachers can observe the instruction of their peers. Teacher educators can also emphasize instructors to attach importance to the constant academic development and critical thinking to enhance their instructional method. Instructors should be directed to be well-informed about instructive issues and take advantage of improved learning chances. They can hold workshops for in-service teachers to encourage them to use technology in education in order to improve their self-efficacy. It is also suggested that teacher educators highlight interaction tools, like mobile applications, which encourage teachers and learners to interact and scaffold that increase self-efficacy. They should develop confidence and competence among in-service teachers to entice learners’ interests and engage them in the learning process. Educational policymakers should hire experienced teachers, as the instructive experience can be an important issue for increasing self-efficacy among teachers. Since technology-supported educational contexts are influential in the improvement of self-efficacy, educational policymakers should provide computer labs, projectors, CD and DVD players for instructors and learners. They should set off learner self-efficacy by holding academic workshops that offer learners and teachers some authentic activities. They can ask learners and teachers to do their best within varied CALL-supported educational contexts. Therefore, the validity of experience, the arrangement of the contexts, and the observation of the learners can result in task difficulty and improvement in learners’ skills. The importance of self-efficacy can motivate consultants to expand their horizons to identify learners’ sources of efficacy and remove obstacles of self-efficacy improvement.

In future work, investigating learner self-efficacy in technology-supported contexts in numerous cultural backgrounds might prove important. Some investigations need to be done on the effect of teacher self-efficacy on learner self-efficacy in online contexts. Furthermore, the relationship between teacher and learner proficiency levels of foreign language and their effect on learner self-efficacy in technology-supported contexts should be considered for the future. Furthermore, case and phenomenological investigations, which provide us the reasons behind learner self-efficacy in technology-supported contexts, are required to be done. The effect of different digital tools on learner self-efficacy needs to be studied. It is also desirable to investigate the impact of technology on improving other language characteristics such as emotional intelligence.

## Author Contributions

The author confirms being the sole contributor of this work and has approved it for publication.

## Conflict of Interest

The author declares that the research was conducted in the absence of any commercial or financial relationships that could be construed as a potential conflict of interest.

## Publisher’s Note

All claims expressed in this article are solely those of the authors and do not necessarily represent those of their affiliated organizations, or those of the publisher, the editors and the reviewers. Any product that may be evaluated in this article, or claim that may be made by its manufacturer, is not guaranteed or endorsed by the publisher.
